# Supplementary material for: Determinants and disparities in skilled birth attendants during childbirth in Bangladesh: A study of machine learning and decomposition analysis
Source: PLoS One. 2026 May 7;21(5):e0346682. doi: 10.1371/journal.pone.0346682 (PMC13152122; doi:10.1371/journal.pone.0346682)
Supplement: S1 Table — (DOCX) [file pone.0346682.s001.docx]

**Table S1: Pseudocode for SHAP implementation and decomposition setup**

| **SHAP implementation** |
| --- |
| **#SHAP CODE for ANN MODEL**  #Run ANN model and required libraries  #Predictor variables is SBA and all column from trainData  set.seed(123)  x_vars <- setdiff(names(trainData), "SBA") #”SBA” the predictor variable  #ANN prediction function for model prediction  ann_predict <- function(X) {  predict(ann_model, X, type = "prob")[, "Yes"]  }  #Wrapper to bypass 'object' argument, Because kernelshap() expects a function with:  pred_fun_ann <- function(object, X) {  ann_predict(X)  }  shap_tb <- kernelshap(  object = NULL,  X = testData[, x_vars],  bg_X = trainData[sample(1:nrow(trainData), 500), x_vars], #random sample of 500  pred_fun = pred_fun_ann) #from training observations  # Converts raw SHAP output into a shapviz object  #For creating plots and further analysis library(shapviz) for shap plots  shap_tb_sv <- shapviz(shap_tb)  # Waterfall plot  sv_waterfall(shap_tb_sv, row_id = 51)  # SHAP beeswarm plot  sv_importance(shap_tb_sv, kind = "beeswarm")  # SHAP Dependency plot  sv_dependence( shap_tb_sv, v = "Wealth_index", color_var = "ANCvisit") +  scale_color_manual(values = c("Inadequate" = "red", "Adequate" = "blue"))  # SHAP feature importance BAR plot  sv_importance(shap_tb_sv, kind = "bar")  # Use library ggplot2 and grid used for design plots |
| **Decomposition Setup** |
| #selected data frame is "df"  df <- df %>%  mutate(weight = V005 / 1e6) # as sampling weight scaled by 6 decimals in BDHS data  # For computing average marginal effects library “margins” is needed  dependent <- "F_SBA"  #one hot coding or dummy variable creation for all explanatory variables  independents <- c( All the created dummy variables excluding the reference one  e,g., "F_devision1","F_devision2","F_devision3","F_devision4","F_devision6", **# F_devision5 is Reference category** "F_devision7","F_devision8")  ses_var <- "V190" # ⇦ SES rank variable (Wealth Index) Poorest to Richest  weight_var <- "weight" # ⇦ survey weight variable  #Regression formula (weighted GLM)  form <- as.formula(paste(dependent, "~", paste(independents, collapse = " + ")))  model <- glm(form, data = df, family = quasibinomial(), weights = df[[weight_var]])  #Prediction of probabilities  df$yhat <- predict(model, type = "response")  # Normalization of weights & Computing fractional rank  df <- df %>%  mutate(w_norm = df[[weight_var]] / sum(df[[weight_var]])) %>%  arrange(.data[[ses_var]]) %>%  mutate(cum_w = cumsum(w_norm),  rank = cum_w - w_norm / 2) # Wagstaff fractional rank  #function for calculating Concentration Index by Formula  ci_w <- function(var, mu = NULL, data = df) {  v <- data[[var]]  if (is.null(mu)) mu <- sum(v * data$w_norm)  cov <- sum(data$w_norm * (v - mu) * (data$rank - 0.5))  2 * cov / mu  }  #calculation of average marginal effect  ame_tbl <- summary(margins(model, variables = independents, type = "response"))  ame_vec <- setNames(ame_tbl$AME, ame_tbl$factor) # named vector  # Weighted Mean of predicted outcome  mu_yhat <- sum(df$yhat * df$w_norm)  # Vectorised stats for every covariate  # weighted mean and CI of each independent variable  mean_x <- sapply(independents, \(v) sum(df[[v]] * df$w_norm))  CI_x <- sapply(independents, ci_w)  # Replace absent AMEs with 0 to avoid NA products  marg_eff <- ame_vec[independents]  marg_eff[is.na(marg_eff)] <- 0  # Elasticity (using AME)  elasticity <- marg_eff * mean_x / mu_yhat  # Contribution  contrib <- elasticity * CI_x  results <- data.frame(  variable = independents,  AME = marg_eff,  mean_x = mean_x,  CI_x = CI_x,  elasticity = elasticity,  contribution = contrib  ) \|>  mutate(pct_of_CI = 100 * contribution / sum(contrib, na.rm = TRUE))  print(results, digits = 4)  #total CI, Explain CI, residual CI  cat("\nTotal CI (predicted SBA): ", round(ci_w("yhat", mu_yhat), 4),  "\nExplained CI (sum contrib): ", round(sum(contrib), 4),  "\nResidual CI: ",  round(ci_w("yhat", mu_yhat) - sum(contrib), 4), "\n")  ##Save results in excel using knit rans kableExtra |
