# Supplementary material for: Determinants and disparities in skilled birth attendants during childbirth in Bangladesh: A study of machine learning and decomposition analysis
Source: PLoS One. 2026 May 7;21(5):e0346682. doi: 10.1371/journal.pone.0346682 (PMC13152122; doi:10.1371/journal.pone.0346682)
Supplement: S2 Table — (DOCX) [file pone.0346682.s002.docx]

**Table S2: Details information about model hyperparameters and required packages**

| **Model names** | **Tuned hyperparameter** | **R library used for training** |
| --- | --- | --- |
| **Logistic Regression (LR)** | — | “stats” |
| **Random Forest (RF)** | mtry = 14 | “randomForest” |
| **Support Vector Machine (SVM)** | sigma = 0.03065403; C = 0.5662576 | “e1071”, “kernlab” |
| **Gradient Boosting Machine**  **(GBM)** | n.trees = 2045; interaction.depth = 10; shrinkage = 0.3313096; n.minobsinnode = 18 | “gbm” |
| **Extreme Gradient Boosting**  **(XGBoost)** | nrounds = 179; maxdepth = 10; eta = 0.3313096; gamma = 4.533342; colsample_bytree = 0.3411699; min_child_weight = 4; subsample = 0.7417793 | “xgboost”, |
| **Decision Tree (DT)** | cp = 0.001285347 | “rpart” |
| **Artificial Neural Network (ANN)** | size = 3; decay = 2.262113 | “nnet” |
| **Naïve Bayes (NB)** | laplace = 0; usekernel = FALSE; adjust = 1 | “naivebayes”, |
| **Light GBM (LGBM)** | Learning_rate= 0.01,  num_leaves=31, max_depth= -1, min_data_in_leaf= 20, verbosity=-1, num of trees= 500 | “lightgbm” |
| **Categorical Boosting (CatBoost)** | Learning_rate= 0.05,  depth= 6, l2_leaf_reg= 1, subsample= 1, num_of_trees= 986 | “catboost” |
| **The model training was performed using software R version 4.5.1** | | |
