# Supplementary material for: Determinants and disparities in skilled birth attendants during childbirth in Bangladesh: A study of machine learning and decomposition analysis
Source: PLoS One. 2026 May 7;21(5):e0346682. doi: 10.1371/journal.pone.0346682 (PMC13152122; doi:10.1371/journal.pone.0346682)
Supplement: Attachment — Submitted filename: Review.docx [file pone.0346682.s003.docx]

Determinants, demographic and socio-economic inequalities in skilled birth attendants during childbirth in Bangladesh: a combined study of machine learning and decomposition analysis

**Manuscript Number: PONE-D-25-44386**

1. The Title of the Manuscript is very long. I suggest making it smaller and compact.
2. At line 199, what is MSBA, its not used elsewhere in paper.
3. The authors did not report the confusion matrix.
4. Since the accuracy is almost nearby for all models. Whether Hyperparameter tuning, cross validation is performed or not?
5. The authors did not report about parameters choice, overfitting or underfitting of model.
6. What were the hyperparameters and how were they tuned?
7. It is unclear whether performance differences between models are statistically significant.
8. It’s not mentioned that after applying SMOTE how many cases are considered for further evaluation. Also, When SMOTE has applied after or before split?
9. A Beeswarm SHAP plot can be added.
10. Its better if authors can provide a research flow and Overview of the machine learning framework applied for better understanding to readers.
11. The conclusion is little longer. It’s better to keep strengths and limitations as separate section.
12. Based on this study what are the possible recommendations.
13. The Abstract is too long, weak Conclusion and Introduction section lacking literature review on ML and XAI applications for SBA prediction. The methodology section did not mentioned utilization of XAI.
14. For the present study, How and why the initial independent variables are selected.
15. A minor English editing is required.
