# Supplementary material for: Determinants and disparities in skilled birth attendants during childbirth in Bangladesh: A study of machine learning and decomposition analysis
Source: PLoS One. 2026 May 7;21(5):e0346682. doi: 10.1371/journal.pone.0346682 (PMC13152122; doi:10.1371/journal.pone.0346682)
Supplement: Attachment — Submitted filename: Response to Reviewers.docx [file pone.0346682.s004.docx]

**Manuscript Number: PONE-D-25-44386**

**Replies to the Reviewers’ Comments**

The authors would like to thank the respected editorial manager and reviewers for providing valuable comments, constructive criticisms and useful suggestions that have greatly improved the quality, presentation and style of the manuscript. The authors have done their level best to address all the comments raised by the respected reviewers in the revised manuscript. The text edited in the revised manuscript is typed in red colour.

The point-by-point replies to the respected editorial manager and reviewers’ comments are as follows:

**Responses to Editorial Manager:**

1. Please ensure that your manuscript meets PLOS ONE's style requirements, including those for file naming. The PLOS ONE style templates can be found at

<https://journals.plos.org/plosone/s/file?id=wjVg/PLOSOne_formatting_sample_main_body.pdf> and

[https://journals.plos.org/plosone/s/file?id=ba62/PLOSOne_formatting_sample_title_authors_affiliations.pdf](https://track.editorialmanager.com/CL0/https:%2F%2Fjournals.plos.org%2Fplosone%2Fs%2Ffile%3Fid=ba62%2FPLOSOne_formatting_sample_title_authors_affiliations.pdf/1/010f019b0b891f7d-747411c6-9e7c-457e-99c2-7b6338f69e40-000000/mGat3Rea-edbpYpZcfgu6t3IAg1MQ-sO0jD9olVJMdQ=241)

**Reply:** Thank you for your valuable suggestion. We formatted the revised manuscript without track change file named “Manuscript” following the submission guidelines of PLOS ONE.

2. Please note that PLOS One has specific guidelines on code sharing for submissions in which author-generated code underpins the findings in the manuscript. In these cases, we expect all author-generated code to be made available without restrictions upon publication of the work. Please review our guidelines at <https://journals.plos.org/plosone/s/materials-and-software-sharing#loc-sharing-code> and ensure that your code is shared in a way that follows best practice and facilitates

reproducibility and reuse.

**Reply:** Thank you for your suggestion. We will be happy and have no issues on sharing the code and the data used in our study.

3. When completing the data availability statement of the submission form, you indicated that you will make your data available on acceptance. We strongly recommend all authors decide on a data sharing plan before acceptance, as the process can be lengthy and hold up publication timelines. Please note that, though access restrictions are acceptable now, your entire data will need to be made freely accessible if your manuscript is accepted for publication. This policy applies to all data except where public deposition would breach compliance with the protocol approved by your research ethics board. If you are unable to adhere to our open data policy, please kindly revise your statement to explain your reasoning and we will seek the editor's input on an exemption. Please be assured that, once you have provided your new statement, the assessment of your exemption will not hold up the peer review process.

**Reply:** Thank you so much for the support. The joint decision made by the authors is that they have no issues for sharing the data publicly.

4. Your ethics statement should only appear in the Methods section of your manuscript. If your ethics statement is written in any section besides the Methods, please move it to the Methods section and delete it from any other section. Please ensure that your ethics statement is included in your manuscript, as the ethics statement entered into the online submission form will not be published alongside your manuscript.

**Reply:** Thank you. We have updated the manuscript adding the ethical statement on the methodology part of the paper.

5. Please include a separate caption for each figure in your manuscript.

**Reply:** Thank you for your valuable suggestion. We have added a separate caption for each figure in the manuscript.

6. If the reviewer comments include a recommendation to cite specific previously published works, please review and evaluate these publications to determine whether they are relevant and should be cited. There is no requirement to cite these works unless the editor has indicated otherwise.

**Reply:** Thank you for your comment. We read the reviewers recommendations very carefully, and they did not mention any specific works for citation.

**Responses to Reviewer 1:**

Recommendations
1) Authors should provide details of hyperparameters, ANN structure, and training methods. If default settings were used, state this explicitly. Ideally, rerun models with tuned parameters and update results.

**Reply:** Thank you. Based on your valuable suggestion we have modified the model’s results. In past default model structure was used. Now we updated the results by tuning best hyperparameter using 5-fold cross validation (Table 4). And we used random search for finding the best hyperparameters with a tune length of 10. Also details about ANN structure has added in the Methodology section.

2) Authors should use k-fold cross-validation or bootstrap methods to report stable averages and confidence intervals for metrics like AUC. Report statistical tests (e.g., DeLong’s test) to check significance of performance differences between models.

**Reply:** Thank you for your suggestion. We have updated the result according to your valuable suggestion. We have used 5-fold CV and reported AUC with 95%CI (Table 4). Also, we have performed DeLong’s Test (Table 5) for checking significance of performance differences between models.

3) Authors should explore SHAP dependence or partial dependence plots to show non-linear relationships. Discuss trade-offs between precision and recall in practical terms (e.g., implications of false negatives in maternal health contexts). Highlight unique contributions of ML beyond regression.

**Reply:** As per your valuable recommendation a SHAP Dependence plot has added (figure 8). The discussion about the model matrices of ANN model have been discussed in maternal health contexts in Discussion part.

4) Authors should provide supplementary code snippets, pseudocode, or detailed appendices describing analytical steps (e.g., specific R/Stata commands). This will allow others to build upon the work and increase transparency.

**Reply:** Thank you. According to your valuable suggestion some commands and name of library functions have added in methodology section.

5) For data samples having missing values, authors should use some imputation techniques, such as SMOTE, or ADASYN.

**Reply:** Thank you for your comment. We also believe that exclusion is not an ideal way. We have a large data set and the variable “number of ANC visit” had 250 women’s information missing (also have missing information on other variables). That’s why we preferred to exclude these sample.

6) The authors should consider experimenting with state-of-the-art models, which may provide stronger predictive performance and deeper insights than the classical methods currently used.

**Reply:** We thank the reviewer for the suggestion. Accordingly, we implemented a state-of-the-art model LightGBM and CatBoost using 5-fold CV with hyperparameters. The LightGBM model achieved accuracy of 0.73, recall of 0.74, specificity of 0.72, precision of 0.83, f1-score of 0.78 and AUC of 0.79, on the other hand CatBoost achieved accuracy of 0.73, recall of 0.75, specificity of 0.69, precision of 0.82, f1-score of 0.78 and AUC of 0.79, which are comparable to the best models in our study.

Due to limitations in the library support within R 4.5.1 the additional advanced models could not be reliably implemented. We have incorporated these results of LightGBM and CatBoost into the revised manuscript and clarified this point in Methodology and Result Section.

7) The manuscript would benefit from thorough proofreading to improve clarity, grammar, and overall readability.

**Reply:** Thank you so much for your comment. Considering your valuable recommendation, we revised the paper and did correction to improve readability.

**Responses to Reviewer 2:**

1. The Title of the Manuscript is very long. I suggest making it smaller and compact.

**Reply:** Thank you. As per your valuable recommendation, we proposed a new title for the manuscript, as follows:

**“Determinants and disparities in skilled birth attendants during childbirth in Bangladesh: a study of machine learning and decomposition analysis”.**

2. At line 199, what is MSBA, it’s not used elsewhere in paper.

**Reply:** Thank you. It was a mistake. Please accept our apologies. The texts are being correctly written.

3. The authors did not report the confusion matrix.

**Reply:** As per your suggestion, we have reported it on methodology part along with how they created. Also, the model metrics which were calculated from model’s confusion matrix of each model. We used test data set for predicting and creation of confusion matrix.

4. Since the accuracy is almost nearby for all models. Whether Hyperparameter tuning, cross validation is performed or not?

**Reply:** Thank you for your suggestion. The authors had taken this recommendation seriously. In past default model structure was used. Now we updated the results by tuning best hyperparameter using 5-fold cross validation. And we used random search for finding best hyperparameters. The results have shown in table 4.

5. The authors did not report about parameters choice, overfitting or underfitting of model.

**Reply:** Thank you. In past default model structure was used. Now we updated the results by tuning best hyperparameter using 5-fold cross validation. And we used random search for finding the best hyperparameters. We justified the performance of model based on AUC value for avoiding overfitting and underfitting of model. The models performances reported in Table 4.

6. What were the hyperparameters and how were they tuned?

**Reply:** Thank you for your comment. In past default model structure was used. Now we updated the results by tuning best hyperparameter using 5-fold cross validation. And we used random search for hyperparameters with a tune length of 10.

7. It is unclear whether performance differences between models are statistically significant.

**Reply:** As per the recommendation of another respected reviewer, we performed DeLong’s test to find out performance difference in between models. The results have shown in Table 5.

8. It’s not mentioned that after applying SMOTE how many cases are considered for further evaluation. Also, When SMOTE has applied after or before split?

**Reply:** Thank you for your comment. We had applied SMOTE to balance the training dataset only and it was applied after splitting the dataset (Details discussed on Methodology section).

9. A Beeswarm SHAP plot can be added

**Reply:** Thank you. A Beeswarm SHAP plot has added according to your suggestion. (Figure 8)

10. Its better if authors can provide a research flow and Overview of the machine learning framework applied for better understanding to readers.

**Reply:** Thank you. Considering your valuable suggestion, a research flow and ML framework is represented in figure 1.

11. The conclusion is little longer. It’s better to keep strengths and limitations as separate section.

**Reply:** As per your valuable recommendation, the authors separated the strength and limitation.

12. Based on this study what are the possible recommendations.

**Reply:** Thank you again for your valuable suggestion. Possible recommendations are added after strength and limitation as a separate section.

13. The Abstract is too long, weak Conclusion and Introduction section lacking literature review on ML and XAI applications for SBA prediction. The methodology section did not mentioned utilization of XAI.

**Reply:** Considering your important recommendation, the authors tried hard to find more literatures of ML application in SBA context. But there were not so many studies found who used ML and SHAP based interpretability in this particular topic. Two published literatures found on African population. (Ref 13 & 32) The Abstract and Conclusion is rewritten based on your suggestion. Also, Details about application of XAI added in the Methodology part.

14. For the present study, How and why the initial independent variables are selected.

**Reply:** Thank you for your comment. We reviewed published literatures on Skilled Birth Attendant. Based on the review we selected the independent variables which mentioned in methodology section.

15. A minor English editing is required.

**Reply:** Thank you for your suggestion. We have read the manuscript thoroughly for searching grammatical mistakes to correct those mistakes. Also, we did try to improve the readability by rewriting some texts.
