# Supplementary material for: Determinants and disparities in skilled birth attendants during childbirth in Bangladesh: A study of machine learning and decomposition analysis
Source: PLoS One. 2026 May 7;21(5):e0346682. doi: 10.1371/journal.pone.0346682 (PMC13152122; doi:10.1371/journal.pone.0346682)
Supplement: Attachment — Submitted filename: Response_to_Reviewers_auresp_3.docx [file pone.0346682.s006.docx]

**Manuscript Number: PONE-D-25-44386R2**

EMID:77c84a23547f9504

**Replies to the Reviewers’ Comments**

The authors would like to thank the respected editorial manager and reviewers for providing valuable comments, constructive criticisms and useful suggestions that have greatly improved the quality, presentation and style of the manuscript. The authors have done their level best to address all the comments raised by the respected reviewers in the revised manuscript. The text edited in the revised manuscript is typed in red colour and **line number** are mentioned based on **“Revised manuscript with track change”** file.

The point-by-point replies to the respected editorial manager and reviewers’ comments are as follows:

**Responses on journal requirements:**

1. Please review your reference list to ensure that it is complete and correct. If you have cited papers that have been retracted, please include the rationale for doing so in the manuscript text, or remove these references and replace them with relevant current references. Any changes to the reference list should be mentioned in the rebuttal letter that accompanies your revised manuscript. If you need to cite a retracted article, indicate the article’s retracted status in the References list and also include a citation and full reference for the retraction notice.

**Reply:** Thank you very much for this important reminder. We have carefully reviewed the reference list and corrected references 3, 9, 16, 17, 61, and 69 to ensure they comply with the required journal style. No references were added or removed during this revision.

**Responses to Reviewer 1:**

Reviewer #1: The authors have addressed the key points raised in my initial review. They strengthened the ML analysis through proper hyperparameter tuning and 5-fold cross-validation, clarified the ANN architecture, and now report performance with confidence intervals and formal model comparisons using the DeLong’s test.

They expanded the interpretability analysis with additional SHAP plots and improved transparency by adding methodological details and supplementary code. While missing data are still handled via exclusion, the rationale is now clearly stated in the manuscript.

**Reply:** Thank you for your positive and encouraging feedback. We appreciate your valuable comments and support, which helped improve the quality of our manuscript.

**Responses to Reviewer 2:**

Recommendations:

1. I suggest retaining abbreviations in the abstract only if they are used within the abstract itself. In the main manuscript, each abbreviation should be defined at its first occurrence, after which the abbreviated form can be used consistently.

Reply: Thank you for your valuable suggestion. We have corrected the use of abbreviation as you recommended (line no 28 to 32 in Abstract) (line no 126, 128, 189, 194, 206, 226, 229, 357, 462 in main texts).

2. It would be beneficial if the authors could provide the code and data used in the study to improve the reproducibility and transparency of the analysis.

Reply: Thank you for your valuable suggestion. To enhance the reproducibility and transparency of our analysis, we have now provided the data, STATA and R code used in our paper on google drive. The data and additional data file for map creation is also available on the following google drive link:

**https://drive.google.com/drive/folders/1eEoIIB3x62POgJvWKMgL8z3pXa2HqgOB?usp=sharing**

3. I suggest moving the detailed parameter information currently presented in Table 4 to the supplementary material to improve the readability of the main text. In addition, the authors should specify the software and libraries used to implement the algorithms, either in the Methods section or in the supplementary material.

Reply: Thank you. Considering your valuable suggestion, we moved the parameter information to supplementary file along with specifying the software (R version 4.5.1) and library names in a separate column (Supplementary Table S2).

4. Kindly improve the language.

Reply: Thank you for your valuable suggestion. We have carefully reviewed the manuscript and made the necessary revisions accordingly to improve the overall language and clarity.

5. Improve the quality of figure 4, 6, 7 and 8. Its better to keep as subplots like A and B. Title can be reduced.

Reply: Thank you for your valuable recommendation. As per your recommendation we have improved the quality of figure 4 and 6 and we made figure 7 and 8 as subplots (a) and (b). Also, title of the figures 4, 7 and 8 have been reduced (line no 548, 568, 588-589, 603-607 in Results section).
